# Association of pre-adolescent adverse childhood experiences with adolescent cholesterol levels: findings from the A-CHILD longitudinal study

**DOI:** 10.3389/fpubh.2026.1745206

**Published:** 2026-04-13

**Authors:** Floret Maame Owusu, Nobutoshi Nawa, Yu Par Khin, Hisaaki Nishimura, Satomi Doi, Aya Isumi, Takeo Fujiwara

**Affiliations:** 1 Department of Public Health, Institute of Science Tokyo, Tokyo, Japan; 2 Department of Global Environmental Health, Institute of Science Tokyo, Tokyo, Japan; 3 Department of Health Policy, Institute of Science Tokyo, Tokyo, Japan

**Keywords:** adolescence, adverse childhood experience, cardiovascular risk, cholesterol, high-density lipoprotein, physical abuse, poverty

## Abstract

**Background:**

Adverse childhood experiences (ACEs) are well known to be associated with poor health outcomes in adulthood. However, its effect on adolescent health remains underexplored, particularly, how cumulative ACE exposure during childhood influences cholesterol levels in adolescence. This study aims to investigate the longitudinal association between cumulative ACE exposure during elementary school and cholesterol levels in middle school using population-based longitudinal study.

**Method:**

We used longitudinal data from the Adachi Child Health Impact of Living Difficulty (A-CHILD) study which followed students from elementary through junior high school in Adachi City, Tokyo from 2015 to 2022 (*N* = 1,460). ACE items (single parenthood, parental history of psychiatric disorders, parental neglect, physical abuse, psychological abuse, witness of domestic violence, and poverty) were measured at each wave in grades 1, 2, 4, and 6. Cumulative ACE was measured by experiencing at least 1 ACE in any of the grades. Cholesterol was measured in grade 8, using blood samples obtained during a school health checkup. We evaluated the association between cumulative ACE and exposure to each ACE with cholesterol levels using linear regression analyses, adjusting for sex, maternal age, parental education, and child behavioral and emotional difficulties.

**Results:**

Out of 1,460 adolescents, 876 (60%) experienced at least 1 ACE during elementary school. Total cumulative effect of ACEs on cholesterol was not observed. Physical abuse and poverty were associated with reduced high-density lipoprotein (HDL) (β = −1.54, 95% confidence interval (CI); −3.07, 0.01 and β = −1.96, 95% CI; −3.34, −0.59, respectively), adjusting for confounders.

**Conclusion:**

Experiencing physical abuse and poverty in elementary school were associated with lower HDL cholesterol in adolescence.

## Introduction

Dyslipidaemia is a major risk factor for ischemic heart disease and stroke globally (1). Evidence suggests that cholesterol level during adolescence is a key predictor of future health, as elevated levels during this period persist in adulthood (2). Emerging research has also revealed that social determinants such as adverse childhood experiences (ACEs) could contribute to altered cholesterol levels, thereby influencing long-term health outcomes (3, 4).

ACEs, defined as stressful or traumatic childhood events, could potentially disrupt normal developmental processes and negatively affect wellbeing (5, 6). A global systematic review indicated that approximately 60% of adults have experienced at least one ACE (7), underscoring its high prevalence. The impact of ACEs on health outcomes, particularly cardiovascular outcomes has been of growing interest (8, 9). For example, a study conducted among depressed adult patients revealed that experiencing physical abuse during childhood was associated with reduced serum total cholesterol levels in adulthood (10). Given the established link between cholesterol and cardiac morbidity (11–13), understanding how ACEs alter cholesterol levels in early life could provide deeper understanding into childhood determinants of cardiovascular risk.

The mechanisms linking ACEs to cholesterol levels may differ between children and adults as children are still undergoing biological, psychological, and social development at that age (14). The hypothalamo-pituitary-adrenocortical (HPA) axis, a primary neuroendocrine stress-response, could be dysregulated following repeated exposure to stress (15). This may result in alteration of glucocorticoid signaling, and increased lipolysis, leading to greater circulating free fatty acids. Increased free-fatty-acid flux to the liver may promote hepatic triglyceride synthesis and secretion of triglyceride-rich lipoproteins contributing to a more atherogenic lipid profile (15, 16). Chronic stress could also engage sympathoadrenomedullary responses, which could further promote lipolysis and reinforce these metabolic changes (16). In addition, children may adapt to stress- related coping behaviors such as poor dietary habits, and lower sleep and physical activity, which could also influence cholesterol production (17, 18). In children, cholesterol is essential for brain development, regulation of cell signaling and hormone synthesis (19–21), all of which are crucial for healthy growth. Disruptions in cholesterol regulation during this stage may therefore lead to lasting health consequences. Notably, recent studies have shown a link between dyslipidaemia and mental health (22, 23), suggesting that dyslipidaemia may be an important biomarker in explaining the association between ACEs and mental health, and may also contribute to the link between ACEs and cardiac morbidity.

Previous studies have investigated the association between ACE and cholesterol outcomes (24–27), however, the findings have been inconsistent. A cross-sectional analysis conducted in the United States among African American and non-Hispanic White adults originally recruited at ages 40–79, found no significant association between ACEs and self-reported hyperlipidaemia (26). In contrast, a longitudinal study conducted among adults originally recruited at 18–30 years in four cities in the United States, which measured ACEs retrospectively at the year 15 follow-up, reported an increased risk of incident hyperlipidaemia among White men who experienced abuse before the age of 18 compared to those who did not (28). One possible reason for these conflicting results could be that most studies have relied on retrospective reporting of ACEs among middle to older-aged adult participants, raising concerns for potential recall bias (24, 26, 28).

To address these limitations, our study uses a prospective, population-based adolescent sample with obtained blood samples to gain a better understanding of the association between ACE and cholesterol outcomes, and focuses on the timing of adolescence, when metabolism of lipid is developing (20, 29). This study therefore aims to investigate the longitudinal association between cumulative ACE exposure during elementary school and cholesterol levels in middle school. Since cholesterol is a modifiable risk factor, findings will provide a foundation for timely interventions to reduce long-term cardiovascular disease burden.

## Methods

### Study design

This study employs an observational longitudinal cohort design using regionally representative data from the Adachi Child Health Impact of Living Difficulty (A-CHILD) study in Adachi City in Tokyo. ACEs were assessed repeatedly via caregiver questionnaires in grades 1, 2, 4 and 6 and cholesterol outcomes (HDL cholesterol (HDL-C) and non-HDL cholesterol (non-HDL-C) were obtained in grade 8 during a health checkup.

### Sample

The first wave began in 2015 and targeted first grade students aged 6–7 years across all 69 elementary schools in Adachi City in Tokyo. Participants were followed up in second grade (7–8 years), fourth grade (9–10 years), sixth grade (11–12 years) and eighth grade (13–14 years, equivalent to second grade junior high).

**Figure 1:**
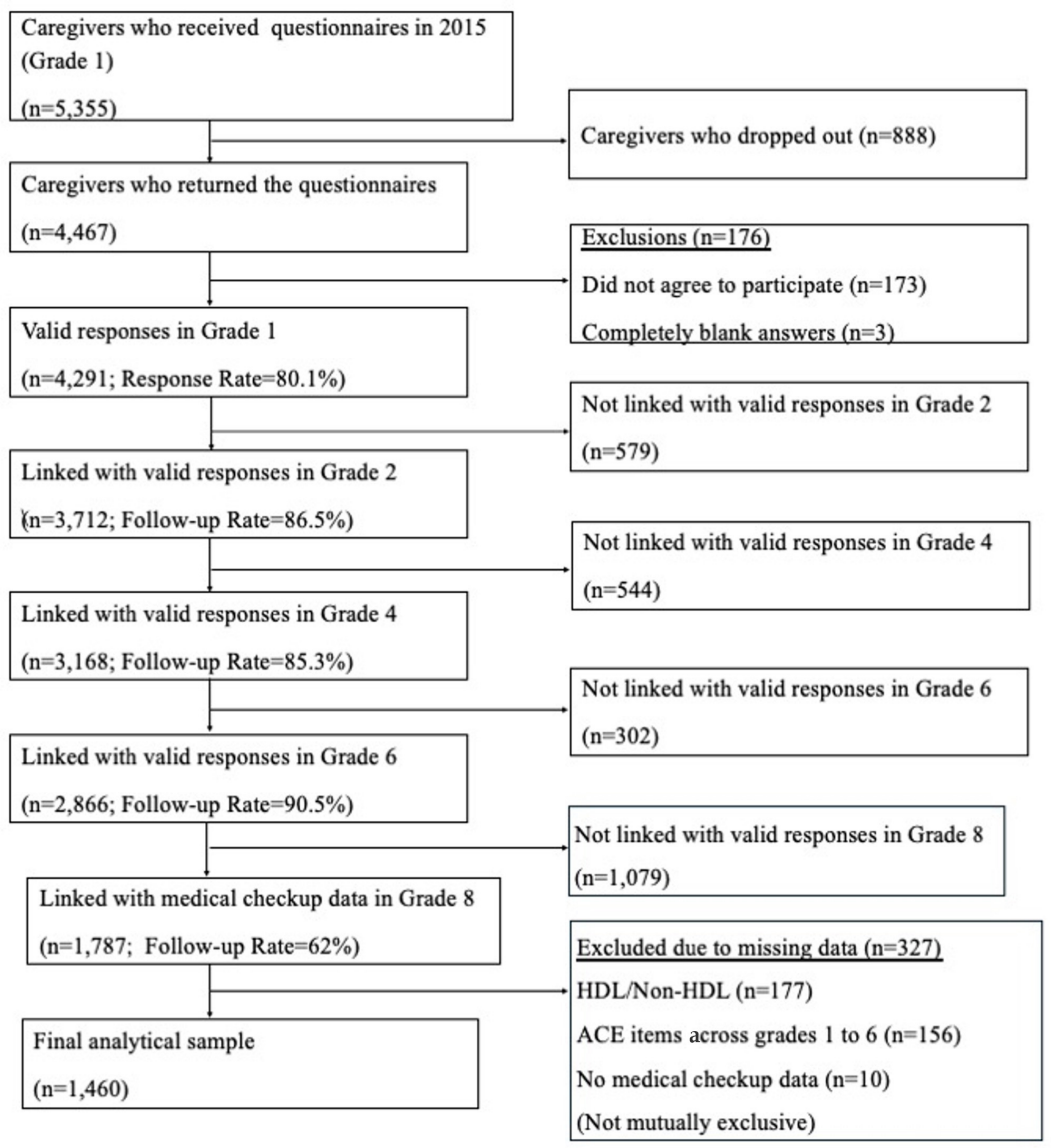
Flow chart of participants.

In the first wave, questionnaires were distributed to children and their caregivers (*n* = 5,355). Questionnaires were completed at home, returned to school and submitted anonymously, resulting in 4,291 valid responses (response rate: 80.1%). The anonymously submitted questionnaires were each assigned a unique study ID by an administrative authority at Adachi City. The research team therefore had access to only de-identified data. For subsequent waves, the data of each survey was linked to this unique study ID. Please see the previously published protocol paper for further details (30). After following up on participants in subsequent waves and linking with medical checkup data in grade 8, 1,787 valid responses were obtained, with a follow-up rate of 62%. After excluding participants with missing exposure and outcome data, the final analytical sample size was 1,460. Informed consent was obtained using an opt-out approach at each grade for survey participation, and at 8th grade for linking the survey with medical checkup data. Details are shown in the flowchart in Figure 1.

This study was approved by the Ethics Committee at the National Center for Child Health and Development (Approval number: 1147) and Tokyo Medical and Dental University (Approval number: M2016-284).

### Measurements

We assessed seven subtypes of ACEs in grades 1, 2, 4 and 6. ACE subtypes were defined based on the classic ACEs study by Felitti et al. (31), that is, single parenthood, parental history of psychiatric disorders, neglect, physical abuse, psychological abuse, witness of domestic violence (DV), and poverty. Due to the sensitivity of the questions, all ACEs were reported by caregivers via self-administered questionnaires, rather than the children. For all ACEs, except poverty, caregivers were asked whether the child had experienced each adversity without specifying a period.

Single parenthood was assessed based on living arrangements with family members, which could be due to death, divorce, or other reasons such as delivery as single mother. Parental history of psychiatric disorders was assessed by asking about history of maternal and paternal psychiatric disorders. Response items used for neglect, physical abuse, psychological abuse, and witness of DV were 1 = “often,” 2 = “sometimes,” 3 = “rarely,” and 4 = “not at all.” Neglect was assessed using two items: “shut the child outside” and “do not feed the child.” Responses were dichotomized, with “rarely,” “sometimes” or “often” categorized as a “yes” response. We coded neglect as 1 (yes) if either of the experiences met this criterion. Physical abuse was assessed using two items: “hit the child's body (buttocks, hand, head, or face)” and “beat the child.” For the former, “often” was categorized as a “yes” response, while for the latter, “rarely,” “sometimes,” or “often” were categorized as a “yes” response. Responses to both items were dichotomized. We coded physical abuse as 1 (yes) when either item was classified as “yes.” Psychological abuse was assessed according to two inquiries, namely, “yell at the child,” in which the responses were dichotomized, with “often” categorized as a “yes” response, and “insult the child repeatedly,” in which the responses were dichotomized with “sometimes” or “often” categorized as a “yes” response. We coded psychological abuse as 1 (yes) when either of the responses was classified as “yes.” Witness of domestic violence was assessed with the question “have a big fight in front of the child,” in which the responses were dichotomized with “sometimes” or “often” which were equated to a “yes” response.

To assess child poverty, caregivers were asked about their annual household income for the last year. They were also asked about material deprivation attributable to financial reasons, experience of payment difficulty of listed activities and items such as school field trips/extracurricular activities and school textbooks over the last year, and whether they received welfare benefits over the past year. We categorized poverty as present if the child met any of the four criteria: (1) annual household income below 3 million JPY, (2) one or more material deprivations, (3) at least one experience of payment difficulty (4) receipt of welfare benefits. This multidimensional assessment of poverty provides a more comprehensive view of the deprivations that children experience (30).

To assess exposure, cumulative ACE was defined as experiencing each ACE at least once across grades 1, 2, 4, or 6. The total numbers of ACEs were categorized as 0, 1, 2, and 3 or more to create ACE scores.

Also, each ACE subtype was coded as exposure occurring at least once across grades 1, 2, 4, or 6. At each wave, caregivers reported whether each ACE occurred during the past year.

### Cholesterol level

HDL-C and non-HDL-C in grade 8 were used as the outcome measures. Total cholesterol and HDL-C were assessed during a school health checkup conducted in 2022. In a laboratory setting, venipuncture blood samples were taken from the arm. The students were not required to fast prior to undergoing the blood tests. Previous studies have supported the use of non-fasting total cholesterol and HDL-C in lipid profile tests (32–34). Both lipid types were measured using an enzymatic method (35). Non-HDL-C was calculated by subtracting HDL-C from total cholesterol. In a longitudinal study among children of mean age 11.9 years followed up for 35 years, childhood non-HDL-C was a better predictor of adult atherosclerotic cardiovascular disease than LDL-C. Other literature have also demonstrated the usefulness of non-HDL-C in dyslipidaemia diagnosis and management (36, 37).

### Covariates

Sex of students (male and female) reported by children at baseline was used. Maternal age, maternal education and paternal education were used as covariates based on previous studies (38–41). The association between ACE with parental age and education has been shown in previous studies (38–41). Similarly, these variables have also been found to be associated with cholesterol (42) and other cardiovascular risk factors (43–45), including obesity and physical inactivity. These cardiovascular risk factors impact cholesterol levels in children and adolescents (46). We also examined child behavioral and emotional difficulties as a mediator as it may exacerbate the association between ACE and cholesterol outcomes due to stress-related dysregulation of physiological and biological metabolism (47).

### Child behavioral and emotional difficulties

Child behavioral and emotional difficulties, measured as total difficulty score (TDS) was derived from the Japanese version of the strength and difficulty questionnaire (SDQ) (48). It assessed emotional symptoms, conduct problems, hyperactivity/inattention and peer problems (49). The score ranges from 0 to 40 with < 13 categorized as normal, 13 to < 16 as borderline, and ≥16 as abnormal/clinical.

### Statistical analysis

Linear regression analysis was conducted to examine the association between cumulative ACE across grades 1 to 6, and cholesterol levels at grade 8. Since HDL-C and non-HDL-C were measured only once in grade 8 as continuous outcomes, which was normally distributed, and cumulative ACEs exposure was defined as being exposed at least once across grades 1, 2, 4, and 6, following previous literature (50), we applied multivariable linear regression. To examine a potential dose–response relationship, we conducted trend analysis by modeling the ACE score (0, 1, 2, and ≥3 ACEs) as an ordinal variable in linear regression and Mann-Kendall score calculation. Further, each ACE subtype was analyzed to assess their individual associations with HDL-C and non-HDL-C.

After analyzing the crude model, the covariates (sex, maternal age, maternal education and paternal education) were adjusted for in Model 1. Multicollinearity was assessed using variance inflation factors (VIFs). VIFs were < 5 for both HDL-C and non-HDL-C, indicating that there was no problematic multicollinearity. Child behavioral and emotional difficulties was further adjusted for in Model 2 to assess potential mediation. All analyses were conducted using the computer software STATA 16 MP for MacOS (STATA Corp., College Station, TX, USA).

## Results

The demographic characteristic of participants is shown in Table 1. There was an approximately equal distribution of males (*n* = 733, 50.2%) and females (*n* = 726, 49.7%) in the sample population. 43.6% (*n* = 636) of mothers had received up to technical school or junior college education while 56.4% (*n* = 823) of fathers had received college or higher education. In addition, 60% (*n* = 876) of children had experienced at least 1 ACE through grade 1 to grade 6. Mean HDL-C and non-HDL-C were 63.7 mg/dL (SD = 12.1) and 106.1 mg/dL (SD = 25.6) respectively.

**Table 1:** Demographic characteristics of participants (*N* = 1,460).

Characteristics		*n* or Mean	% or SD
HDL cholesterol (mg/dL)		63.7	12.1
Non-HDL cholesterol (mg/dL)		106.1	25.6
Child's sex	Male	733	50.2
	Female	726	49.7
Maternal age (years old)		31.4	8.3
Maternal education	High school or lower	518	35.5
	Technical school/Junior College	636	43.6
	University or higher	278	19.0
	Others/missing	28	1.9
Paternal education	High school or lower	566	38.8
	Technical school/Junior College	304	20.8
	University or higher	519	35.6
	Others/missing	71	4.9
^*^SDQ-TDS	Normal	1,091	74.7
	Borderline	190	13.0
	Clinical	176	12.1
	Missing	3	0.2
ACE Score	0	584	40.0
	1	441	30.2
	2	237	16.2
	3+	198	13.6

^*^Strengths and difficulties questionnaire (total difficulties score).

Table 2 shows the dose-response association of ACEs during elementary school years, with HDL-C and non-HDL-C levels in grade 8. In the crude model, compared to experiencing no ACEs, experiencing one or two ACEs was not associated with non-HDL-C (1 ACE: β = 0.19, 95% CI; −2.98, 3.36; 2 ACEs: β = 0.37, 95% CI; −3.50, 4.24) and HDL-C (1 ACE: β = −0.25, 95% CI; −1.75, 1.25; 2 ACEs: β= −1.43, 95% CI; −3.26, 0.40). Similar trends were observed in model 1 which adjusted for sex, maternal age, maternal education and paternal education, and model 2 which included child mental health difficulties. Experiencing three or more ACEs was associated with lower HDL-C levels in the crude model (β = −2.57, 95% CI; −4.53, −0.62), but this association became non-significant in model 1(β = −1.69, 95% CI; −3.67, 0.29) or model 2 (β = −1.42, 95% CI; −3.49, −0.64). The p for trend between cumulative ACE exposure and cholesterol levels was significant only for HDL-C in the crude model (*p*-value = 0.01) but was not significant in models 1 and 2. For non-HDL-C, no significant association was observed across all models. Similarly, Kendall's tau indicated a significant negative trend between ACE score and HDL-C (tau-b = −0.051, p = 0.01), but no evidence of trend for non-HDL-C (tau-b = 0.001, *p* = 0.95).

**Table 2:** Association between adverse childhood experiences (ACEs) and cholesterol levels (*N* = 1,460).

ACE	CRUDE		MODEL 1		MODEL 2	
	Non-HDL	HDL	Non-HDL	HDL	Non-HDL	HDL
	Co-efficient (95% CI)	Co-efficient (95% CI)	Co-efficient (95% CI)	Co-efficient (95% CI)	Co-efficient (95% CI)	Co-efficient (95% CI)
Total ACE 0	ref	ref				
1	0.19 (−2.98, 3.36)	−0.25 (−1.75, 1.25)	0.06 (−3.04, 3.16)	−0.15 (−1.63, 1.32)	−0.10 (3.22, 3.03)	−0.07 (−1.56, 1.42)
2	0.37 (−3.50, 4.24)	−1.43 (−3.26, 0.40)	0.10 (−3.75, 3.96)	−1.16 (−3.00, 0.68)	−0.17 (−4.08, 3.74)	−1.01 (−2.87, 0.85)
3+	1.97 (−2.17, 6.10)	**−2.57 (−4.53**, −0.62)^*^	3.01 (−1.15, 7.17)	−1.69 (−3.67, 0.29)	2.50 (−1.84, 6.83)	−1.42 (−3.49, 0.64)
*P for trend*	0.25	**0.01** ^*^	0.11	0.09	0.19	0.19

Trend analysis was conducted by modeling the ACE score (0, 1, 2, and ≥3 ACEs) as an ordinal variable in linear regression and Mann–Kendall score calculation. Confidence interval, CI. MODEL 1-Adjusted for sex, maternal age, maternal education and paternal education. MODEL 2-Adjusted for sex, maternal age, maternal education, paternal education and child behavioral and emotional difficulties.

^*^Indicates *p*-value < 0.05. Bold values indicate statistical significance (*p* < 0.05).

The association between each subtype of ACEs (single parenthood, parental history of psychiatric disorders, neglect, physical abuse, psychological abuse, witness of DV, and poverty) and cholesterol levels using linear regression analysis is shown in Table 3. Having parents with a history of psychiatric disorders was associated with reduced HDL-C levels in the crude model (β= −2.30, 95% CI; −4.30, −0.30), with no association observed in models 1 and 2. There was no association with non-HDL-C across all models. Experiencing physical abuse from parents was associated with reduced HDL-C levels in the crude model (β = −1.98, 95% CI; −3.53, −0.43) and in model 1(β = −1.54, 95% CI; −3.07, −0.01). When child behavioral and emotional difficulties was further adjusted for in Model 2 to assess potential mediation, no significant association was observed, suggesting potential mediating role of child behavioral and emotional difficulties on the association between experiencing physical abuse from parents and HDL-C levels. Poverty was consistently associated with reduced HDL-C levels across all models: the crude model (β = −2.27, 95% CI; −3.59, −0.94), model 1(β = −1.96, 95% CI; −3.34, −0.59) and model 2 (β = −1.86, 95% CI; −3.25, −0.46). In contrast, it was associated with increased non-HDL-C levels only in the crude model, but no significant association remained after adjusting for confounders. Single parenthood, neglect, psychological abuse, and witness of DV were neither associated with HDL-C nor non-HDL-C levels.

**Table 3:** Association between ACE subtypes and cholesterol levels (*N* = 1,460).

^†^ACE subtypes	CRUDE		MODEL 1		MODEL 2	
	Non-HDL	HDL	Non-HDL	HDL	Non-HDL	HDL
	Co-efficient (95% CI)	Co-efficient (95% CI)	Co-efficient (95% CI)	Co-efficient (95% CI)	Co-efficient (95% CI)	Co-efficient (95% CI)
Single Parenthood	3.69 (−0.34, 7.71)	−0.75 (−2.66, 1.16)	4.05 (−0.49, 8.59)	0.23 (−3.67, 0.29)	3.84 (−0.74, 8.41)	0.40 (−1.78, 2.58)
Parental history of psychiatric disorders	2.67 (−1.55, 6.89)	**−2.30 (−4.30**, −0.30)^*^	3.31 (−0.82, 7.44)	−1.72 (−3.69, 0.24)	2.95 (−1.28, 7.18)	−1.54 (−3.56, 0.47)
Neglect from parents	−2.35 (−5.75, 1.04)	−0.15 (−1.76, 1.46)	−0.64 (−3.97, 2.69)	0.25 (−1.34, 1.83)	−0.98 (−4.35, 2.39)	0.41 (−1.20, 2.01)
Physical abuse from parents	−0.31 (−3.60, 2.97)	**−1.98 (−3.53**, −0.43)^*^	0.68 (−2.53, 3.89)	**−1.54 (−3.07**, −0.01)^*^	0.32 (−2.97, 3.61)	−1.38 (−2.94, 0.19)
Psychological abuse from parents	1.31 (−3.34, 5.96)	0.62 (−1.59, 2.82)	1.68 (−2.85, 6.21)	0.74 (−1.41, 2.91)	1.23 (−3.39, 5.85)	1.07 (−1.13, 3.27)
Witness of domestic violence between parents	−0.16 (−4.66, 4.33)	0.45 (−1.68, 2.58)	−0.55 (−4.93, 3.83)	0.56 (−1.53, 2.64)	−0.86 (−5.28, 3.55)	0.75 (−1.35, 2.86)
Poverty	**2.88 (0.08, 5.69)** ^*^	**−2.27 (−3.59,−0.94)** ^*^	2.70 (−0.20, 5.59)	**−1.96 (−3.34**, −0.59)^*^	2.54 (−0.39, 5.47)	**−1.86 (−3.25**, −0.46)^*^

^†^ACE subtypes indicate exposure reported at least once across grades 1, 2, 4, or 6. CI, Confidence interval. MODEL 1-Adjusted for sex, maternal age, maternal education and paternal education. MODEL 2–Adjusted for sex, maternal age, maternal education, paternal education and child mental health difficulties.

^*^Indicates *p*-value < 0.05. Bold values indicate statistical significance (*p* < 0.05).

Supplementary Table 1 shows the association between participants included in the analytic sample and those not included. Baseline characteristics differed between participants included in the analytic sample and those not included, particularly for parental education and SDQ-TDS (Supplementary Table 1).

## Discussion

To the best of our knowledge, this study is the first to examine the association between ACEs and objectively measured cholesterol levels using a prospective longitudinal study design in an adolescent population. We found that there was no association between cumulative ACE exposure during elementary school years and cholesterol levels in middle school. However, exposure to poverty and physical abuse in pre-adolescence were associated with lower HDL-C levels in early adolescence, with child behavioral and emotional difficulties potentially mediating the association between physical abuse and reduced HDL-C level.

Our finding showed that ACE exposure is not associated with corresponding cholesterol levels among adolescents. While previous studies in adult populations have reported positive dose-response associations between ACEs and adult diseases, such as obesity, hypertension and mental health (51–53), we did not find similar evidence for cholesterol levels in early adolescents. Our finding addresses a significant gap in research and suggests that the pathway linking ACEs to cholesterol levels is distinct in adolescence, potentially influenced by specific ACE subtypes, and individual biological sensitivity. Childhood and adolescence are crucial stages of development as early-life experiences influence neuron differentiation and synapse formation which control psychological and physiological regulation (14). Notably, children vary in their biological responses to stress, affecting cortisol response, and subsequently influencing cholesterol production (54, 55). This physiological variability could account for the absence of the dose-response association observed in our study.

We also found that experiencing poverty was associated with reduced HDL-C levels, which has been observed in previous studies (56, 57). Poverty in childhood is a chronic stressor that exposes children to material deprivation and impacts all aspects of the child's life including food, education and social development (58). Children living in poverty are more likely to engage in unhealthy behaviors such as poor dietary habits, sedentary lifestyle and smoking (59, 60), all of which have been reported to be associated with low HDL-C levels (61). These risky behaviors formed in childhood become habits which persist into adolescence and increase the risk of having altered cholesterol levels over time. In addition, chronic stress from childhood poverty could result in dysregulation of the hypothalamic-pituitary-adrenal (HPA) axis, contributing to suppression of HDL-C production (15). The interplay of behavioral and biological pathways of poverty could explain its association with reduced HDL-C levels.

Additionally, we found that physical abuse was associated with reduced HDL-C levels, with child behavioral and emotional difficulties serving as a potential mediator. This suggests that the pathway linking physical abuse to reduced HDL-C levels could be partially explained by mental health difficulties in children. Among the ACEs examined in this study, physical abuse is uniquely exerted directly on the child, potentially resulting in more severe psychological consequences. Child physical abuse has been found to be associated with internalizing and externalizing mental health difficulties either indirectly through pathways such as insecure attachment, unhealthy behavioral patterns, poor coping strategies, and social isolation (62–67), or directly as reported in previous studies (68–71). Mental health difficulties have also been associated with reduced HDL-C levels (72–74), suggesting a potential pathway linking physical abuse to reduced HDL-C levels. Furthermore, physical abuse in children has been linked to reduced choline levels in adolescents (75), which is a nutrient essential for synthesis of phosphatidylcholine, a major phospholipid component of HDL-C (76, 77). This reduction in choline levels may impair phosphatidylcholine production and thereby contribute to low HDL-C production.

Our study has notable strengths. One is the use of extracted blood samples for measuring cholesterol levels, which ensures objective measurement of biomedical data. Another is the prospective longitudinal study design which minimizes the risk of recall bias related to ACEs and strengthens causal inference. Our study also has some limitations; first, this study was conducted among elementary school students in Adachi City in Tokyo and so results may not be generalizable to children and adolescents living in other regions and of different age ranges. Second, parents' self-reporting of ACEs may lead to underreporting due to social desirability or fear of disclosure. This could result in exposure misclassification, likely attenuating the association between ACEs and cholesterol. Future studies should consider multiple informants, including child self-report, where appropriate. Third, including a culturally diverse population in future studies would help to understand the cultural context of ACE on health outcomes. Fourth, this study only focused on cholesterol, and so future studies should assess ACE exposure in relation to a broader set of cardiometabolic outcomes. Cholesterol was also assessed at a single time point, which could result in within-person biological variability and random measurement error, reducing precision and attenuating the associations. Also, differences between included and non-included participants suggest possible selection bias, which could impact the strength of the association. Lastly, covariates such as dietary patterns and physical activity were not included as covariates in the analyses. Although these factors are associated with cholesterol levels, they were not considered confounders of the ACE–cholesterol association and may also lie on the causal pathway. However, residual confounding such as genetic factors cannot be fully excluded. Future studies that examine the timing of ACEs in childhood that influences health outcomes in adolescence would be informative. Finally, formal mediation analysis will be required in future studies to confirm child behavioral and emotional difficulties as a possible mediator between ACEs and cholesterol levels.

Despite the limitations, this study contributes to the body of evidence on early-life risk factors for cardiovascular disease. The implication of this study highlights the importance of early identification and prevention of ACEs, specifically physical abuse, as well as alleviation of childhood poverty. It also provides evidence that interventions supporting mental health needs among children exposed to ACEs may help prevent early alterations in cholesterol levels, potentially reducing long-term cardiovascular risk. Based on these findings, physicians could recognize ACEs as potential early life risk factors for unfavorable cholesterol levels, particularly reduced HDL-C levels. Thus, comprehensive psychosocial and socio-economic history could be incorporated as part of routine adolescent health screenings, such as school health checkups or clinical visits, to identify at-risk children.

## Conclusion

In this longitudinal prospective study, we found that there was no dose-response association between number of ACEs and HDL-C and non-HDL-C. Poverty and physical abuse were however associated with reduced HDL-C levels, with child behavioral and emotional problems being a potential mediator between ACEs and physical abuse. Findings from this study are beneficial in informing childhood interventions targeted at reducing cardiovascular risks.

## Data Availability

The datasets presented in this article are not publicly available due to ethical restrictions. The data are available on request from the corresponding author. Requests should be directed to fujiwara.hlth@tmd.ac.jp.
