# Supplementary material for: Association of pre-adolescent adverse childhood experiences with adolescent cholesterol levels: findings from the A-CHILD longitudinal study
Source: Front Public Health. 2026 Apr 13;14:1745206. doi: 10.3389/fpubh.2026.1745206 (PMC13111572; doi:10.3389/fpubh.2026.1745206)
Supplement: Supplementary file 1 [file Table_1.docx]

Association of Pre-adolescent Adverse Childhood Experiences with Cholesterol Levels in Adolescence: Findings from the A-CHILD Longitudinal Study

Floret Maame Owusu^a^, Nobutoshi Nawa^b^, Yu Par Khin^a^, Hisaaki Nishimura^a^, Satomi Doi^c^, Aya Isumi^c^, Takeo Fujiwara^a*^

^a^ Department of Public Health, Institute of Science Tokyo, Tokyo, Japan

^b^ Department of Global Environmental Health, Institute of Science Tokyo, Tokyo, Japan

^c^ Department of Health Policy, Institute of Science Tokyo, Tokyo, Japan

Address for correspondence: Takeo Fujiwara, Department of Public Health, Institute of Science Tokyo, M&D Tower 16F, 1-5-45 Yushima, Bunkyo-ku, Tokyo 113-8510, Japan (e-mail: fujiwara.hlth@tmd.ac.jp; Tel.: +81-3-5803-5188).

Supplementary Table 1: Baseline characteristics of participants included in the final analytic sample versus those not included (N=6,076)

| Characteristics |  | Included (N=1,460) | | Not included (N=4616) | | P-value |
| --- | --- | --- | --- | --- | --- | --- |
|  |  | **n or Mean** | **% or SD** | **n or Mean** | **% or SD** |  |
| Child’s sex | Male | 733 | 50.2 | 1,464 | 51.8 | 0.33 |
|  | Female | 726 | 49.8 | 1,362 | 48.2 |  |
| Maternal age (years old) |  | 38.4 | 4.9 | 38.0 | 5.2 | 0.01 |
| Maternal education | High school or lower | 518 | 35.5 | 1,064 | 23.1 | <0.001 |
|  | Technical school /Junior College | 636 | 43.6 | 1,069 | 23.2 |  |
|  | University or higher | 278 | 19.0 | 592 | 12.8 |  |
|  | Others/missing | 28 | 1.9 | 1,891 | 41.0 |  |
| Paternal education | High school or lower | 566 | 38.8 | 1,013 | 22.0 | <0.001 |
|  | Technical school /Junior College | 304 | 20.8 | 437 | 9.5 |  |
|  | University or higher | 519 | 35.6 | 1,061 | 23.0 |  |
|  | Others/missing | 71 | 4.9 | 2,105 | 45.6 |  |
| *SDQ-TDS | Normal | 1,178 | 80.7 | 1,291 | 28.0 | <0.001 |
|  | Borderline | 134 | 9.2 | 191 | 4.14 |  |
|  | Clinical | 131 | 9.0 | 245 | 5.31 |  |
|  | Missing | 17 | 1.2 | 2,889 | 62.6 |  |

*Strengths and Difficulties Questionnaire (Total Difficulties Score)
